# Same-day antiretroviral therapy initiation for HIV-infected adults in South Africa: Analysis of routine data

**DOI:** 10.1371/journal.pone.0227572

**Published:** 2020-01-14

**Authors:** Rivka R. Lilian, Kate Rees, James A. McIntyre, Helen E. Struthers, Remco P. H. Peters

**Affiliations:** 1 Anova Health Institute, Johannesburg, South Africa; 2 School of Public Health and Family Medicine, University of Cape Town, Cape Town, South Africa; 3 Division of Infectious Diseases & HIV Medicine, Department of Medicine, University of Cape Town, Cape Town, South Africa; 4 School of Public Health, University of the Witwatersrand, Johannesburg, South Africa; 5 Department of Medical Microbiology, School of Public Health & Primary Care (CAPHRI), Maastricht University Medical Centre, Maastricht, The Netherlands; Yeshiva University Albert Einstein College of Medicine, UNITED STATES

## Abstract

Same-day initiation (SDI) of antiretroviral therapy (ART) has been recommended to improve ART programme outcomes in South Africa since August 2017. This study assessed implementation of SDI over time in two South African districts, describing the characteristics of same-day initiators and evaluating the impact of SDI on retention in ART care. Routine data were analysed for HIV-infected adults who were newly initiating ART in Johannesburg or Mopani Districts between October 2017 and June 2018. Characteristics of same-day ART initiators were compared to later initiators, and losses to follow-up (LTFU) to six months were assessed using Kaplan Meier survival analysis and multivariate logistic regression. The dataset comprised 32 290 records (29 964 from Johannesburg and 2 326 from Mopani). The overall rate of SDI was 40.4% (n = 13 038), increasing from 30.3% in October 2017 to 54.2% in June 2018. Same-day ART initiators were younger, more likely to be female and presented with less advanced clinical disease than those initiating treatment at later times following diagnosis (p<0.001 for all). SDI was associated with disengagement from care: LTFU was 30.1% in the SDI group compared to 22.4%, 19.8% and 21.9% among clients initiating ART 1–7 days, 8–21 days and ≥22 days after HIV diagnosis, respectively (p<0.001). LTFU was significantly more likely among clients in Johannesburg versus Mopani (adjusted odds ratio (aOR) = 1.43, p<0.001) and among same-day versus later initiators (aOR = 1.45, p<0.001), while increasing age reduced LTFU (aOR = 0.97, p<0.001). In conclusion, SDI has increased over time as per national guidelines, but there is serious concern regarding the reduced rate of retention among same-day initiators. Nevertheless, SDI may result in a net programmatic benefit provided that interventions are implemented to support client readiness for treatment and ongoing engagement in ART care, particularly among younger adults in large ART programmes such as Johannesburg.

## Introduction

South Africa’s antiretroviral therapy (ART) programme for the treatment of human immunodeficiency virus (HIV) infection was launched in 2004. At the time, 4.1 million adults over the age of 15 years were living with HIV and within a decade, an additional 2.2 million adults were infected with the virus [1]. National ART guidelines evolved over this time to treat increasing numbers of HIV-infected clients and to provide improved care and treatment services. South Africa’s ART programme has since grown to be the largest world-wide, with an estimated 4.2 million adults receiving ART in 2017 [2].

When South Africa’s ART programme first started, adults with CD4 counts <200 cells/mm^3^ were eligible to initiate treatment [3]. The CD4 cut-off was raised to 350 cells/mm^3^ in August 2011 [4] and to 500 cells/mm^3^ from January 2015 [5]. However, despite the expanding ART eligibility criteria, South Africa faced challenges in reaching the second 90-90-90 target, namely the provision of ART to 90% of HIV diagnosed individuals, with only 56.9% of HIV-diagnosed adults receiving ART nationally by the middle of 2015 [6]. In September 2016 the National Department of Health (NDoH) implemented universal test and treat (UTT) whereby all adults became eligible to initiate ART irrespective of CD4 count [7], but adult ART coverage increased only minimally to 61% in 2017 [2]. This is consistent with findings from a South African study regarding the significantly decreased likelihood of ART initiation with increasing baseline CD4 count [8], as clients feel “too healthy” to initiate treatment [9]. In addition, numerous other barriers to ART initiation may have impacted treatment coverage rates, including distance to testing centres, transport costs, over-busy clinics and the need for repeat facility visits at the time ART eligibility was being assessed [10, 11].

In order to address ongoing challenges with ART initiation, the NDoH implemented same-day initiation (SDI), namely ART initiation on the same day as HIV diagnosis [12]. The NDoH circular signed in August 2017 requested all public health facilities to scale up ART initiation for all HIV-infected individuals as per UTT guidelines, with an emphasis on providing SDI for individuals newly diagnosed with HIV who were clinically and psychologically ready for lifelong ART [12]. Clinical readiness encompasses screening for symptoms of tuberculosis (TB) and cryptococcal meningitis, as initiation of ART must be delayed in clients with these conditions in order to avoid complications such as immune reconstitution inflammatory syndrome [13]. When appropriately implemented, SDI has the potential to prevent the loss of ART-eligible clients from pre-ART care prior to treatment initiation, a challenge that has been described in multiple sub-Saharan African settings, including South Africa [14, 15]. A number of randomised trials of rapid ART initiation have indeed demonstrated multiple benefits of rapid initiation compared to later ART start, including improved ART uptake by 3 months, increased retention in care at 12 months, higher rates of 12-month viral suppression and reduced risk of mortality [16–19]. However, randomised trials precisely control the environment in which the intervention is being assessed, and it therefore cannot be automatically assumed that findings from these trials regarding the benefits of SDI would translate into routine settings [20]. Although a number of observational studies of SDI in sub-Saharan African settings have also been performed, these studies focussed on pregnant and breastfeeding women and the results may therefore not be generalizable to the wider adult population [21–24]. Other routine studies have been limited to hospitals [25] or have described SDI in the context of specialised interventions such as peer-delivered linkage case management or a revised ART initiation counselling model implemented at a single facility [26, 27]. There is thus a paucity of data regarding the implementation of a national policy for SDI across the whole adult population in routine sub-Saharan African settings. In order to fill this gap, this study aimed to analyse routine programme data to assess implementation of SDI over time and its impact on programme outcomes in an urban and rural district of South Africa. Specifically, this study aimed to compare characteristics of clients who initiate ART on the same day as HIV diagnosis to clients who initiate treatment at later time points after diagnosis, and to assess the impact of SDI on retention in ART care.

## Methods

### Data source and study population

A single data source was used for this analysis, namely Three Interlinked Electronic Registers.Net (TIER.Net), an electronic ART database developed by the University of Cape Town’s Centre for Infectious Disease Epidemiology and Research [28]. TIER.Net is used operationally in public health facilities in South Africa to monitor baseline clinical care and client outcomes over time, and is also the platform into which HIV tests are electronically captured. Client characteristics and demographic information are routinely captured into TIER.Net by staff working at the healthcare facilities. This study analysed routine TIER.Net data from clients initiating ART in Johannesburg District in Gauteng Province and Mopani District in Limpopo Province. These districts were chosen as Anova Health Institute had access to Johannesburg and Mopani TIER.Net data owing to Anova’s work in these districts as the designated United States Agency for International Development (USAID) district support partner, and as such, comprise a convenience sample. Johannesburg, an urban district, has a population density of 3 018.4 people/km^2^ with an estimated medical scheme coverage of 25.0%, compared to rural Mopani District which has a population density of 59.8 people/km^2^ and medical scheme coverage of 6.7% [29]. Antenatal HIV prevalence, a proxy for overall HIV prevalence, is 29.6% in Johannesburg and 24.5% in Mopani [30], with 50.4% and 73.7% of clients remaining on ART in the respective HIV programmes in 2017 [29].

For the purposes of this study, January 2019 data were extracted from TIER.Net, including client demographics, particulars of ART treatment and programme outcomes. Records were included in the analysis for HIV-infected adults aged 15 to 80 years who were newly initiating ART at primary healthcare facilities in Johannesburg or Mopani District between October 2017 and June 2018 (n = 56 284). This time period was chosen to allow time for distribution of the August 2017 NDoH circular [12] and to allow for a minimum follow-up period of six months. Records were excluded where there was no electronic record of a positive HIV test in TIER.Net (23 358 (41.5%)) and where there were data quality concerns regarding missing or nonsensical dates (636 (1.1%)). Data quality concerns included a recorded HIV test date after the ART start date, a missing outcome date for clients who died, were lost to follow-up (LTFU) or who transferred from one facility into another, or an outcome date occurring prior to the ART start date. The final dataset thus comprised 32 290 records (29 964 from Johannesburg and 2 326 from Mopani).

### Statistical analysis

Time to ART initiation was calculated from the date of the positive HIV test to the ART start date. Where these dates were the same, clients were classified as same-day initiators. Later initiators, namely clients who initiated ART 1 or more days after the HIV test date, were divided into three groups: clients who initiated ART 1 to 7 days, 8 to 21 days or ≥22 days after HIV diagnosis. The 1 to 7-day initiation group reflects clinically uncomplicated clients, the 8 to 21-day group reflects ART initiation in clients with co-morbidities such as TB, and the ≥22-day group reflects delayed treatment initiation in clients who either lost contact with the health system or had serious co-morbidities such as cryptococcal meningitis [13]. The proportion of same-day initiators was plotted over time. Characteristics of same-day initiators were compared to later initiators using Kruskal-Wallis equality-of-populations rank test for continuous variables and chi-squared (*χ*^2^) or Fisher’s exact tests for categorical variables, as appropriate. Programme outcomes were assessed six months after ART initiation and classified as active in care, LTFU (namely clients without drug in hand for more than 90 days as defined in TIER.Net), died or transferred out. Characteristics of clients who were LTFU by six months were assessed using univariate and multivariate logistic regression.

Kaplan Meier survival analysis was used to compare LTFU in same-day ART initiators versus later initiators. Follow-up time was defined as the time between ART initiation and outcome date for clients who died, were LTFU or transferred out, and as the time between ART initiation and the TIER.Net export date for clients who remained active in care. Where clients had a follow-up time of zero (i.e. ART initiation date and outcome date were the same), a follow-up time of half a day was assigned. Follow-up time was administratively censored at six months after ART initiation. Clients who died or transferred out were censored at the date of death or date of transfer, respectively. Survival curves were compared using a Peto-Peto-Prentice test for equality of survivor functions, which is not affected by differences in censoring patterns across groups and is appropriate even when hazard functions are not proportional [31].

Analyses were performed using Microsoft Excel 2010 and Stata version 14.2 (StataCorp LLC, College Station, Texas, USA). A p-value of < 0.05 was considered significant.

### Ethical consideration

The study was approved by the Human Sciences Research Council Research Ethics Committee (REC 3/22/08/18). This study analysed anonymised TIER.Net data that were routinely collected at healthcare facilities for monitoring purposes. Individual client consent was therefore not required, as no data collection was performed for the purposes of this study and no client files or electronic medical records were accessed at any stage.

## Results

### Trends in same-day initiation over time

**Fig 1:**
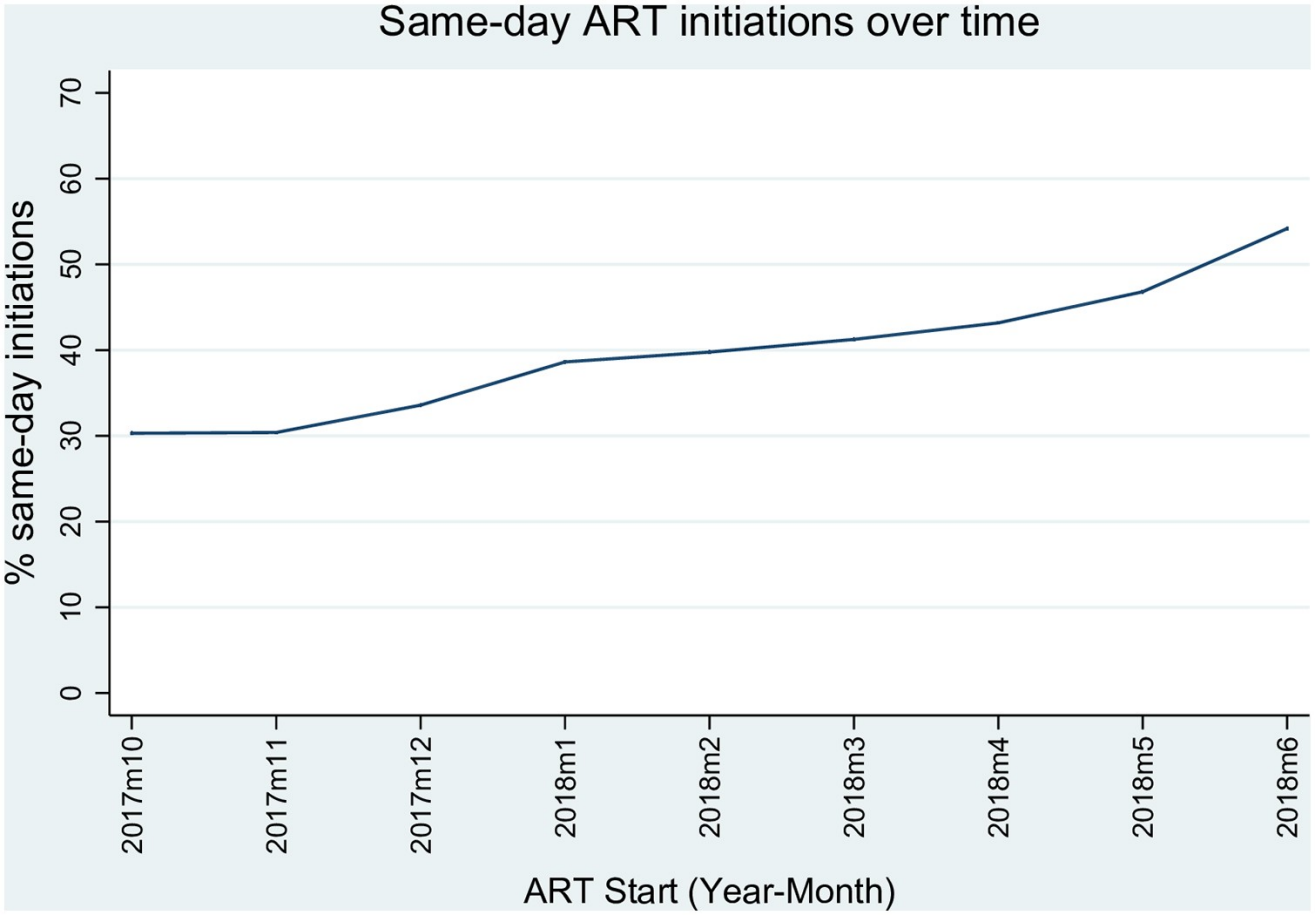
Trends in same-day antiretroviral therapy initiation over time. (ART, antiretroviral therapy).

In October 2017, 30.3% of clients starting ART in Johannesburg and Mopani districts were initiated on treatment on the same day as their HIV test (Fig 1). The proportion of same-day initiators increased over time, reaching 54.2% by June 2018. The overall rate of SDI from October 2017 to June 2018 was 40.4% (n = 13 038) and was equivalent in Johannesburg and Mopani districts (40.3% and 41.1%, respectively; p = 0.461).

### Characteristics and outcomes of clients by time to antiretroviral therapy initiation

Same-day ART initiators were younger (p < 0.001) and more likely to be female (p < 0.001) compared to clients initiating ART at later times after HIV diagnosis) (Table 1). Same-day initiators had less advanced HIV-infection than later initiators, presenting with higher baseline CD4 counts and a significantly higher proportion of World Health Organization (WHO) stage I and II disease compared to stage III and IV disease (p < 0.001 for both). Among females aged 15–50 years, those who initiated ART at later times after HIV diagnosis were significantly less likely to be pregnant (p < 0.001). Of those females who were pregnant at ART start, 84.1% initiated ART on the same day as HIV testing compared to 32.9% among females who were not pregnant at ART start. Same-day initiation was associated with being LTFU from the ART programme by six months after treatment initiation (p < 0.001), with a lower proportion of deaths among same-day initiators (p < 0.001) (Table 2). Among clients who died, 38.0% initiated ART more than 22 days after HIV diagnosis.

**Table 1:** Baseline characteristics of clients initiating antiretroviral therapy by time from HIV diagnosis to treatment initiation.

	Same-day	1–7 days	8–21 days	22+ days	P-value
n^a^	13 038	7 261	6 534	5 457	
Site					**0.012**
Jhb District	12 082 (92.7%)	6 747 (92.9%)	6 022 (92.2%)	5 113 (93.7%)	
Mopani District	956 (7.3%)	514 (7.1%)	512 (7.8%)	344 (6.3%)	
Gender					**< 0.001**
Male	3 375 (25.9%)	2 816 (38.8%)	2 685 (41.1%)	2 359 (43.2%)	
Female	9 663 (74.1%)	4 445 (61.2%)	3 849 (58.9%)	3 098 (56.8%)	
Age at ART start (years), median (range)	31.9 (15.1–79.6)	34.8 (15.3–75.7)	35.8 (15.0–79.4)	35.4 (15.0–78.2)	**< 0.001**
Baseline CD4<100					**< 0.001**
Yes	1 118 (14.1%)	1 045 (19.0%)	1 296 (25.3%)	1 044 (26.9%)	
No	6 805 (85.9%)	4 446 (81.0%)	3 820 (74.7%)	2 836 (73.1%)	
WHO stage at ART start					**< 0.001**
I/II	11 673 (94.3%)	6 344 (91.4%)	5 227 (82.5%)	3 893 (74.6%)	
III/IV	702 (5.7%)	596 (8.6%)	1 110 (17.5%)	1 329 (25.5%)	
Pregnant at ART start (females 15–50 years)					**< 0.001**
Yes	4 601 (50.0%)	439 (10.8%)	165 (4.8%)	265 (9.5%)	
No	4 608 (50.0%)	3 611 (89.2%)	3 256 (95.2%)	2 530 (90.5%)	

ART, antiretroviral therapy; HIV, human immunodeficiency virus; Jhb, Johannesburg; WHO, World Health Organization

Data are n (%) unless otherwise indicated. Total value differs between variables because of missing data.

Statistically significant differences are shown in bold.

^a^ Same-day, 1–7 day, 8–21 day and 22+ day initiations represent 40.4%, 22.5%, 20.2% and 16.9% of the study sample, respectively.

**Table 2:** Programme outcomes by time from HIV diagnosis to initiation of antiretroviral therapy.

	Same-day	1–7 days	8–21 days	22+ days	P-value^a^
n	13 038	7 261	6 534	5 457	
Programme outcomes					
Active	8 399 (64.4%)	5 276 (72.7%)	4 860 (74.4%)	3 976 (72.9%)	**< 0.001**
LTFU	3 804 (29.2%)	1 586 (21.8%)	1 254 (19.2%)	1 162 (21.3%)	**< 0.001**
Died	42 (0.3%)	37 (0.5%)	48 (0.7%)	78 (1.4%)	**< 0.001**
TMO	793 (6.1%)	362 (5.0%)	372 (5.7%)	241 (4.4%)	**< 0.001**

HIV, human immunodeficiency virus; LTFU, lost to follow-up; TMO, transferred or moved out

Data are n (%). Statistically significant differences are shown in bold.

^a^ *χ*^2^ test for trend across time to ART initiation groups.

### Impact of same-day initiation on retention in care

**Fig 2:**
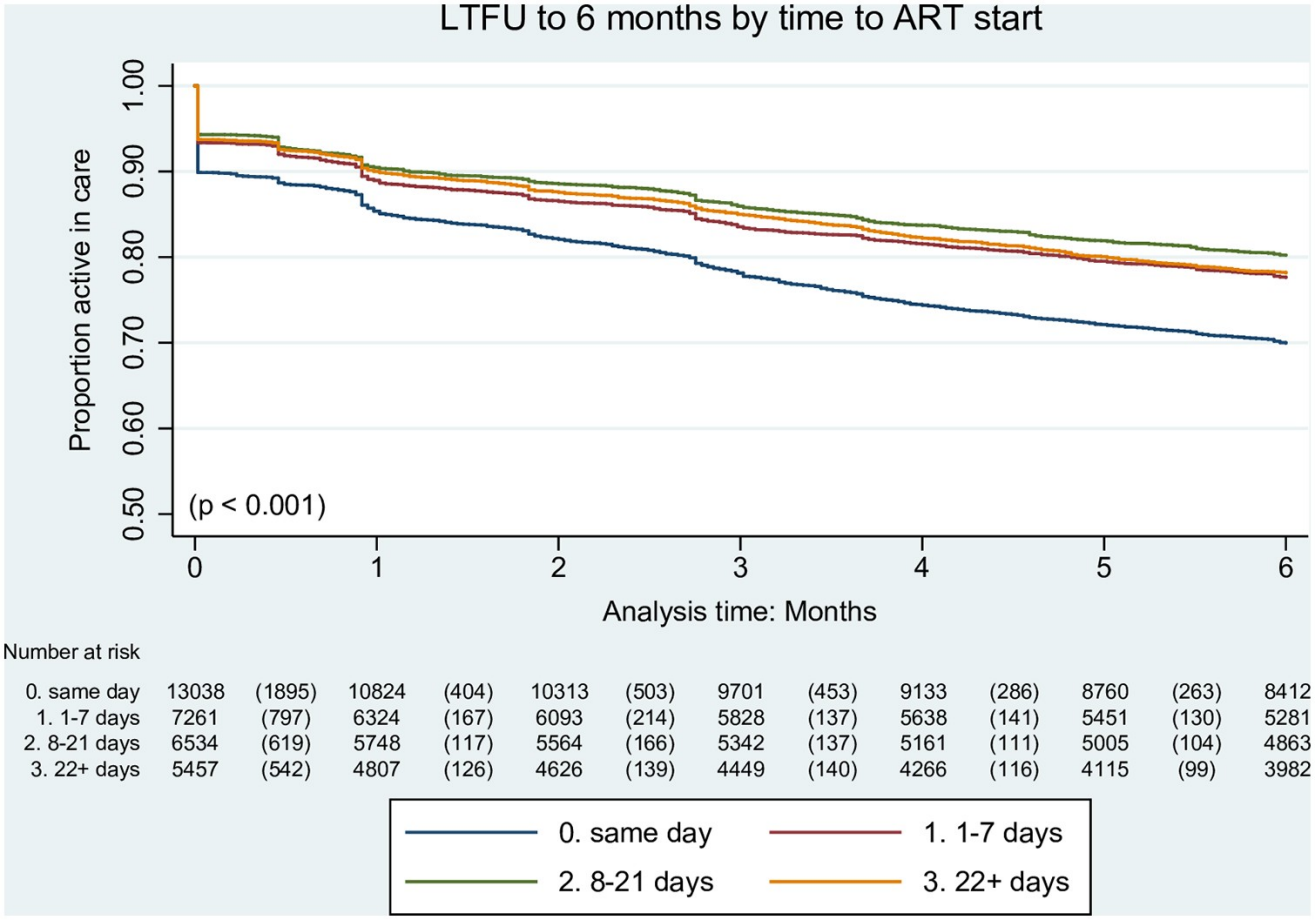
Kaplan-Meier survival curve of LTFU by time from HIV diagnosis to antiretroviral therapy initiation. (p = Peto-Peto test for equality of survivor functions. ART, antiretroviral therapy; HIV, human immunodeficiency virus; LTFU, loss to follow-up).

Programme loss was higher among clients with same-day ART initiation compared to clients who initiated treatment at later time points after HIV diagnosis (LTFU of 30.1% in the SDI group compared to 22.4%, 19.8% and 21.9% among clients initiating ART 1–7 days, 8–21 days and ≥22 days after HIV diagnosis, respectively (p < 0.001)) (Fig 2). When the three later initiator groups were combined, the additional programme loss at six months among same-day initiators compared to clients initiating ART ≥1 day after HIV diagnosis was 8.7% (30.1% versus 21.4%, respectively, p < 0.001). Losses in the immediate period after treatment initiation are clearly evident among all clients initiating ART, but particularly in the SDI group.

Clients who were LTFU from the ART programme by six months were more likely to have received care in Johannesburg District than in Mopani District (odds ratio (OR) 1.49, p < 0.001), to have WHO stage I or II disease compared to stage III or IV disease (OR 1.14, p = 0.002) and to have initiated ART on the same day as HIV testing as opposed to later ART initiation (OR 1.60, p < 0.001) (Table 3). The odds of LTFU was less likely among males (OR 0.79, p < 0.001) and older clients (OR 0.96, p < 0.001). In multivariate analysis, LTFU remained significantly more likely among clients in Johannesburg and those with SDI, with a 40–45% increased odds of being lost from the programme (adjusted OR (aOR) 1.43, p < 0.001 for Johannesburg versus Mopani and aOR 1.45, p < 0.001 for SDI versus later initiation). The odds of LTFU also remained significantly less likely among older clients (aOR 0.97, p < 0.001).

**Table 3:** Characteristics of clients lost to follow-up from the ART programme by six months after treatment initiation.

			Unadjusted OR		Adjusted OR^a^	
	Active	LTFU	OR (95% CI)	P-value	OR (95% CI)	P-value
n	22 511	7 806				
Site						
Jhb District	20 776 (92.3%)	7 391 (94.7%)	1.49 (1.33–1.66)	**< 0.001**	1.43 (1.27–1.60)	**< 0.001**
Mopani District	1 735 (7.7%)	415 (5.3%)	Ref		Ref	
Gender						
Male	8 122 (36.1%)	2 411(30.9%)	0.79 (0.75–0.84)	**< 0.001**	0.97 (0.91–1.03)	0.269
Female	14 389 (63.9%)	5 395 (69.1%)	Ref		Ref	
Age at ART start (years), median (range)	34.7 (15.0–79.6)	31.5 (15.4–79.0)	0.96 (0.96–0.97)	**< 0.001**	0.97 (0.96–0.97)	**< 0.001**
Baseline CD4<100						
Yes	3 245 (19.9%)	910 (18.8%)	0.93 (0.86–1.01)	0.073	-	-
No	13 028 (80.1%)	3 937 (81.2%)	Ref			
WHO stage at ART start						
I/II	18 969 (88.0%)	6 627 (89.3%)	1.14 (1.05–1.24)	**0.002**	0.92 (0.85–1.01)	0.073
III/IV	2 600(12.1%)	797 (10.7%)	Ref		Ref	
Same-day ART initiation						
Yes	8 399 (37.3%)	3 804 (48.7%)	1.60 (1.52–1.68)	**< 0.001**	1.45 (1.37–1.53)	**< 0.001**
No	14 112 (62.7%)	4 002 (51.3%)	Ref		Ref	

ART, antiretroviral therapy; CI, confidence interval; Jhb, Johannesburg; LTFU, lost to follow-up; OR, odds ratio; Ref, reference; WHO, World Health Organization. Data are n (%) unless otherwise indicated. Total value differs between variables because of missing data. Statistically significant differences are shown in bold.

^a^ Multivariate logistic regression adjusting for site, gender, age, WHO clinical stage and same-day ART initiation.

Among 7 806 clients who were LTFU, a third (2 515, 32.2%) were lost on the same day as ART initiation, i.e. these clients did not return to care after the ART initiation visit. The proportion of losses on the same day as ART initiation among clients who were LTFU was significantly higher among same-day ART initiators compared to later initiators (34.7% (n = 1 319) compared to 29.9% (n = 1 196), respectively; p < 0.001).

## Discussion

This study of routine ART data in a rural and urban district of South Africa demonstrates an increasing trend in the proportion of clients initiated on ART on the same day as HIV diagnosis, in line with national recommendations for SDI [12]. Same-day ART initiation is acceptable to the vast majority of clients assessed in randomised trials (98–99%) [17, 18] and is also well accepted in high-income settings (>90%) [32]. However, uptake of SDI is less consistent in low- and middle-income settings where the study was not a randomised trial. Among pregnant women, same-day ART initiation in South Africa varied between 20% and 91% under Prevention of Mother-to-Child Transmission (PMTCT) Programme Option A (ART eligibility defined as CD4 count ≤ 350 cells/mm^3^), and in Malawi, uptake depended on the level of integration of antenatal care and HIV/ART services (19% uptake in sites where ART was not integrated and 57% uptake in fully integrated sites) [21, 22, 24]. A study of routine data in 16 Zimbabwean hospitals demonstrated SDI in 65% of newly diagnosed patients [25], in line with the 54% uptake of SDI in June 2018 in this study. It is important to bear in mind that uptake of SDI is not only dependant on client readiness, but also on clinical criteria, as initiation of ART needs to be delayed in clients presenting with symptoms of TB or cryptococcal meningitis [13]. In the RapIT trial in South Africa, the most common reason for delaying SDI was the need for TB treatment or confirmation of TB or a WHO stage III/IV condition [19]. In addition, in our experience, implementation of SDI is also influenced by attitudes of healthcare workers and acceptability of SDI to the treating clinician.

If SDI rates are to be improved beyond the 54% uptake found in this study, clients to be targeted for SDI need to be characterised. Later ART initiators in this study were found to be older than same-day initiators and were more likely to be male. Two previous studies found no significant difference in age between same-day and later ART initiators, however one study was performed in pregnant women which is in itself age-limiting and the second was performed in only 86 individuals in San Diego, majority of whom were men who have sex with men, and is therefore not comparable to the South African adult population in this study [22, 33]. An analysis of same-day ART initiators in Zimbabwe also found no significant association between age or gender and SDI, though only hospitals were included and the sample size was smaller than in this study (628 same-day initiators versus 13 038 in this study) [25]. In our setting it is expected that later initiators are older and male, as these clients are known to present later for ART care with lower baseline CD4 counts [34, 35] which puts them at risk of advanced clinical disease that delays ART initiation. Men and older clients have similarly been found to present late for care in other sub-Saharan African countries [36], highlighting the need to target men and older clients for early ART initiation if SDI is to be successfully scaled up. In addition, later ART initiators in this study were found to have more advanced HIV-infection in terms of baseline CD4 count and WHO stage, which is to be expected in view of recommendations to defer ART initiation in ill clients [13]. With regard to pregnant women, South Africa prescribes PMTCT programme Option B+, defined as lifelong ART for all women, irrespective of CD4 count, with immediate treatment initiation on the day of diagnosis [5]. SDI was implemented in > 80% of females who were pregnant at ART start in this study, in line with this guideline. In Zimbabwe, the high rate of SDI among clients testing for HIV at maternal and child health clinics was attributed to the intensive training, advocacy and community sensitisation associated with the rapid roll-out of Option B+ [25]. Similar training and advocacy for the wider adult population will likely be needed if SDI is to be successfully scaled up across the whole ART programme.

There have been conflicting findings regarding the association between SDI and programme loss in studies to date. A number of randomised trials have found significantly improved retention in care among clients randomised to rapid or same-day ART initiation procedures [17–19]. However, the controlled nature of such trials may limit their generalisability to routine settings [20] and other studies have indeed found differing results. Some studies have demonstrated no difference in programme loss between clients with rapid versus delayed ART initiation [22, 32], while other studies in both pregnant and non-pregnant adults have found significantly increased LTFU or a trend to increased LTFU with SDI [21, 23, 37, 38], in agreement with findings in this study from both the survival analysis and the multivariate regression, which demonstrated a 45% increased odds of LTFU with SDI. Interestingly, one of the trials that demonstrated an overall increase in retention with rapid ART initiation did in fact find a higher rate of LTFU after ART start in the rapid initiation versus standard care arm (17% versus 8%, respectively); however, pre-initiation losses were much lower in the rapid initiation arm (3% versus 28%, respectively) which offset the post-initiation LTFU [19]. Thus, while SDI may improve linkage to ART care by reducing pre-ART LTFU, it appears to shift programme losses to later in the HIV care cascade. In order to outweigh the excess post-initiation LTFU associated with SDI, which in this study amounted to 8.7% at six months, efforts should focus on improving ART initiation rates and supporting client readiness for ART. Improving ART initiation requires interventions to address both structural- and individual-level barriers to ART access, including establishing more ART sites, improving clinic operations, strengthening caregiver training and addressing financial barriers through travel cost reimbursement schemes [39, 40]. With regard to client readiness, WHO’s good practice statement that calls for ‘people-centred care’ should be followed, namely, clients must be supported to make an informed decision regarding when to start ART, with SDI only being implemented in individuals who indicate their willingness to do so [41]. Furthermore, it is important to acknowledge that SDI is not a short-cut for adherence counselling and continued support must be provided to same-day ART initiators even after treatment has been initiated [13]. Support and counselling is particularly important on the day of treatment initiation given the high proportion of early programme losses associated with SDI, with 35% of same-day initiators who are LTFU not returning to the ART programme after the initiation visit.

In addition to the significant association with SDI, programme loss in this study was also predicted by younger age, in agreement with previous findings [42–46], and by ART treatment in Johannesburg District. The higher rate of LTFU in Johannesburg versus Mopani may, at least in part, be explained by the large size of the Johannesburg programme, as rate of programme expansion and larger programme size have been shown to be significantly associated with increased LTFU [47]. This may be attributed to inferior quality of care and counselling at large facilities where there is a trade-off between quantity and quality [47]. Ongoing efforts to improve quality of care and adherence counselling, particularly at larger sites, are warranted.

This study analysed TIER.Net data from adults accessing treatment in South Africa’s ART programme, which is the largest world-wide. TIER.Net is a longitudinal patient-level dataset that is used routinely in public health clinics and it was therefore possible to perform a robust analysis on a large sample. However, this study has a number of limitations. Firstly, in order to properly assess the impact of SDI on ART care, the number of clients successfully engaged in care through same-day and later ART initiation would need to be compared to the number of clients who did not return for ART initiation following a positive HIV test, as it is these clients in particular who may have benefited from SDI. Since this dataset only includes clients who initiated ART, the impact of SDI on improving initiation of ART could not be assessed. Secondly, baseline TB data were not available in TIER.Net and the impact of TB on delayed ART initiation could therefore not be evaluated, nor could TB be adjusted for in the multivariate analysis investigating predictors of LTFU. Thirdly, missing data necessitated the exclusion of multiple records from the analysis. In particular, positive HIV tests were poorly captured; however, we do not believe that this impacted findings regarding the proportion of SDI or its influence on treatment outcomes. Fourthly, this analysis included only two districts in South Africa, representing a convenience sample from areas in which Anova Health Institute was working at the time. Findings from this study should therefore be generalised to other settings with caution. Finally, this study was limited to adult clients and no conclusions can be drawn regarding the impact of SDI on retention in ART care among infants and children.

## Conclusions

In conclusion, this study demonstrates increasing implementation of SDI over time in a rural and urban district of South Africa in line with national guidelines. There is serious concern regarding the reduced rate of retention among same-day ART initiators, particularly in the short term, and interventions to support client readiness for treatment are therefore essential. In addition, ongoing support and counselling for clients who have already initiated ART through SDI are required, in particular for younger adults initiating treatment in larger programmes. The increased linkage to ART care potentially associated with same-day treatment initiation may result in a net programmatic benefit from SDI, provided that wider programme improvements are implemented. Continued monitoring of outcomes in routine settings will be required in order to accurately determine the overall programmatic impact of SDI.

## Supporting information

S1 Table Same-day initiation dataset. (XLSX)
